# P128: Improving hand hygiene compliance in a teaching hospital

**DOI:** 10.1186/2047-2994-2-S1-P128

**Published:** 2013-06-20

**Authors:** R Cocconi, L Arnoldo, M Dal Cin, P Del Giudice, R Fabro, A Faruzzo, D Tignonsini, S Brusaferro

**Affiliations:** 1 Direzione Medica di Presidio, Azienda Ospedaliero-Universitaria Santa Maria della Misericordia, Italy; 2 Department of Medical and Biological Sciences, University of Udine, UDINE, Italy

## Introduction

Although WHO project “Clean care is safer care” was introduced many years ago in our hospital, Hand Hygiene (HH) practice shows low compliance level in some wards at increased risk of Healthcare Associated Infections (HAI).

## Objectives

The project objective is to determine the causes of HH non-compliance and to identify targeted solutions for improvement.

## Methods

The project was carried out with the collaboration of Joint Commission Center for Transforming Healthcare, using TST© methodology, from April to September 2012. We chose to implement the project in a pilot ward, an internal medicine ward.

We selected two groups of observers. The first ones, the secret observers, had the mandate to see whether the individual washed his or her hands upon entering and exiting the room. The second group had trained to be just-in-time (JIT) coaches. The JIT coaches approached health care workers who are found to not be washing hands appropriately and query them as to the reasons for their non-compliance (non observable contributing factors).

## Results

After 3 weeks of data collection experience, with secret observers, the baseline HH compliance in the pilot unit was an average of 26.7%. Entering room was worse than exit room, 22.4% versus 31,7%.

Non-compliance contributing factors were: improper use of gloves (57.3%), frequently entry or exit room (15.5%), hands full of supplies (7.8%), follow person in exit or entry room (6.8%) and equipment shared (5.8%).

Non observable contributing factors, coming from JIT coaches, were: perception that HH is not necessary (50.0%), distracted (41.7%) and skin irritation (8.3%).

After one month of targeted solution implemented, such us: relocation of glove dispensers in the ward, standardize work processes, use a code word for distracted people, etc. HH compliance rose to an average of 65% with a gain of 34,7%.

## Disclosure of interest

None declared

